# Synergistic Effect between Alcohol Consumption and Familial Susceptibility on Lung Cancer Risk among Chinese Men

**DOI:** 10.1371/journal.pone.0040647

**Published:** 2012-07-16

**Authors:** Lap Ah Tse, Ignatius Tak-sun Yu, Xiao-rong Wang, Hong Qiu, Joseph Siu Kie Au

**Affiliations:** 1 Division of Occupational and Environmental Health, Jockey Club School of Public Health and Primary Care, The Chinese University of Hong Kong, Hong Kong SAR, China; 2 Department of Clinical Oncology, Queen Elizabeth Hospital, Kowloon, Hong Kong SAR, China; Baylor College of Medicine, United States of America

## Abstract

We aimed to examine the effect of alcohol consumption on lung cancer risk stratified by smoking, and to explore whether the impact of alcohol was modified by familial susceptibility to cancer. We recruited 1208 male lung cancer incident cases and 1069 community referents during 2004–2006 and collected their lifetime history of alcohol consumption, cigarette smoking, and family cancer history. Unconditional multivariate logistic regression analysis was performed to estimate the adjusted odds ratio (OR). We tested multiplicative-scale interaction between exposures of interest and examined the additive-scale interaction using synergy index. A moderate association between frequent alcohol consumption and lung cancer was observed among men who had family cancer history (OR = 4.22, 95%CI: 2.46–7.23) after adjustment of smoking and other confounders, while the alcohol effect among men without family history was weak (OR = 1.24, 95%CI: 0.95–1.63) and it became no excess in the never smokers. We observed a consistent synergistic effect between alcohol drinking and family cancer history for all lung cancers and the adenocarcinoma, while there was no multiplicative-scale interaction between the exposures of interest (likelihood ratio test for interaction, p>0.05). Our study revealed a possible synergistic effect between alcohol consumption and familial susceptibility for lung cancer risk; however, this observed possible association needs to be confirmed by future larger analytic studies with more never smoking cases.

## Introduction

Lung cancer is the leading cause of cancer death in both men and women worldwide with cigarette smoking being the predominant risk factor [1]. However, the fact that only a small fraction of smokers have developed lung cancer and there are many cases among never smokers suggests that genetic susceptibility, diet and other environmental risk factors (e.g., asbestos, environmental tobacco smoke [ETS], alcohol consumption) may play roles in lung cancer etiology [2]–[5]. Familial aggregation of cancer has been found to be associated with 71% (95% confidence interval [95% CI]: 49–96%) excess risk of lung cancer in men and 73% (95% CI: 50–100%) in women compared with those without familial susceptibility [6]. Such relationship, however, might not be entirely attributable to the genetic variations and the contributions from shared environmental factors could have played a role [7]–[11]. Alcohol consumption, a behavior that is frequently co-present with cigarette smoking, has been shown to have a positive association with lung cancer but the evidence remains controversial mainly due to the residual confounding effect of cigarette smoking [5]. A more recent study pooled 7 prospective studies of 399,767 participants (with 3,137 lung cancer cases) demonstrating that heavy alcohol drinking (≥30 grams per day) was associated with a non-significantly increased risk of lung cancer in men (relative risk [RR]  = 1.21, 95% CI: 0.91–1.61) and women (RR = 1.16, 95% CI: 0.94–1.43) [12], and their findings were consistent with the previous findings [5].

Alcohol drinking was proposed to increase the risk of lung cancer through the alcohol metabolite acetaldehyde which is a known carcinogen classified by the International Agency of Research on Cancer [13]. It is logical to hypothesize that the carcinogenic effect of alcohol may be enhanced among subjects with family cancer history (a proxy of genetically determined susceptibility) who may have inherent limitations of DNA repair to assaults of environmental risk factors including alcohol consumption; hence, the interaction between genetic susceptibility and alcohol consumption is likely to occur [14]. Using data from a large case-referent study conducted in Chinese males, we examined the effect of alcohol consumption on the risk of lung cancer, stratified by smoking status. We also explored whether the effect of alcohol consumption on lung cancer risk was modified by familial susceptibility to cancer.

## Materials and Methods

Study methods of this large case-referent study have been described elsewhere [4], [15]–[18]. Briefly, we consecutively recruited 1208 histologically-confirmed new cases of primary lung cancer among males aged 35–79 years from the largest oncology centre in Hong Kong during Feb 2004 - September 2006; 1069 Chinese male referents without physician-diagnosed cancer in any site were randomly selected through residential telephone directories from the same districts of the cases, with frequency matching to cases in 5-year age groups. The ethics committees of both the Chinese University of Hong Kong and Queen Elizabeth Hospital approved the study protocol (KC/KE 04-0014/ER-1) and the interview procedures for both the lung cancer cases and community referents. Specifically, we obtained written informed consent for all the lung cancer cases in this study; we obtained verbal consent for the community referents who agreed to be interviewed by telephone (about 70%) while the written informed consent was only obtained for those who participated in the face-to-face interview (about 30%).

Using a structured questionnaire, trained interviewers carried out personal interviews to collect each participant’s information on cigarette smoking [16], alcohol consumption, family history of cancer, and other potential confounding factors including socio-demographics, residential indoor air pollution (i.e., exposure to radon [19], [20], incense burning [17], use of mosquito coils, and years of cooking by frying), ETS exposures in the workplace and household since childhood [4], exposures to the confirmed or suspected occupational carcinogens [14], [18], dietary habits, and past history of lung diseases. Smoking was classified as never (smoking <20 packs of cigarettes in his lifetime or <1 cigarette a day for 1 year) [21], former (quitted smoking ≥ 2 years) [22], and current (still smoking or quit <2 years) smoking. We asked every current and former smoker about their daily consumptions of cigarette, years of smoking, and years since cessation (if quit). We quantified lifetime cigarette smoking in pack-years (1 pack = 20 cigarettes). Participants were instructed to report whether they had consumed any of these alcohol beverages (beer, red wine, white including rice wine, and liquor) for at least one year. If the answer was ‘yes’, then the frequency [<1 day per month, 1–3 days per month, 1–3 days per week, and ≥4 days per week] was asked; these frequencies were further regrouped into two categories to improve the statistical power because very few participants drank alcohol ≥4 days per week: occasional (<1 day/week) and frequent users (≥1 day/week). However, we did not collect the quantity of alcohol intake (i.e., grams per day) from the participants. We asked the subjects whether their 1^st^ degree relatives (i.e., natural mother, father, or siblings) had ever been diagnosed by a doctor of having lung cancer or cancer of other sites. Positive family history of cancer meant that either lung cancer or cancer of other sites had ever been diagnosed in any of the 1^st^ degree relatives.

Unconditional multivariate logistic regression models were performed to estimate the odds ratio (OR) and the 95% confidence interval (95% CI) using the following strategies. We initially included various potential confounding factors into a ‘base’ model using a forward stepwise method among all subjects, and the variables that were finally retained in the ‘base’ model were age at interview, place of birth, education level, residential radon exposure, past history of lung diseases, intake of non-orange related fruit, smoking status and smoking pack-years. Results from one of our companion papers showed that cigarette smokers is a strong risk factor for lung cancer with an OR of 12.16 (95% CI: 9.34–15.84) for current smokers and 3.11 (95% CI: 2.38–4.07) for former smokers compared with those who had never smoked [16].

We performed stratified analyses according to the status of smoking (never, former, current) to elucidate the role of cigarette smoking for the association between alcohol and lung cancer. We further examined the independent and joint effect of alcohol consumption and family history of cancer (negative, positive) on lung cancer risk. We tested possible interactions between exposures of interest on the multiplicative-scale by including a product term (i.e., likelihood ratio test for interaction, p<0.05). We examined interactions between exposures of interest on the additive-scale (i.e., risk difference modifications) by the synergy index (SI) following an approach proposed by Hosmer and Lemeshow [23]. If the synergy index was significantly above one, the joint effect would conform to an additive-scale interaction [23], [24]. Separate analyses were only performed in the 440 adenocarcinoma cases (89 never smokers, 124 former smokers, and 227 current smokers) because of too few never smoking cases of squamous and small cell lung cancers (n = 5 and 0 respectively).

## Results

A total of 1201 lung cancer cases and 997 community referents who had complete information on family cancer history were included in the multivariate data analyses. Overall, the mean age of lung cancer cases (65.8±9.5 years) at the time of interview was fairly similar to that of the community referents (66.2±9.9 years, p>0.05). Compared with the referents, lung cancer cases were more likely to be ever smokers (89% vs. 50%) and ever alcohol users (62.6% vs. 48.4%), and they had smoked heavier (pack-years: 49.58±31.82 vs. 35.00±33.15) and consumed alcohol for a relatively longer duration (28.5±15.0 years vs. 25.2±15.6 years).

As shown in Table 1, there were more lung cancer cases (239, 19.8%) than the community referents (134, 12.5%) who had at least one first-degree relative with any cancers (p<0.001). The OR for family cancer history derived from a main effect multivariate model was 2.28 (95% CI: 1.69–3.08), and it tended to higher for those with family lung cancer history (2.72, 95% CI: 1.71–4.33) than those with a family history of other cancers (2.07, 95% CI: 1.45–2.96). The multivariate adjusted OR for the frequent alcohol users was 1.38 (95% CI: 1.08–1.76, vs. never users), however, there was no evidence for an excess risk of lung cancer for the occasional users (OR = 1.06, 95% CI: 0.81–1.38) (Table 1).

**Table 1:** Distributions of alcohol consumption and family cancer history by status of case and referent, the odds ratios (OR) and 95% confidence interval (95% CI) of lung cancer among Hong Kong Chinese men, 2004–2006^a^.

Levels of exposure	Referents(n = 1069)	Cases(n = 1208)	Odds ratioand 95% CI
Alcohol consumption			
Never	541 (50.6)	445 (36.8)	1.00
Occasional	242 (22.6)	269 (22.3)	1.06 (0.81, 1.38)
Frequent	276 (25.8)	487 (40.3)	1.38 (1.08, 1.76)
Family cancer history			
Overall cancers	134 (12.5)	239 (19.8)	2.28 (1.69, 3.08)
Lung cancer	47 (4.4)	96 (7.9)	2.72 (1.71, 4.33)^b^
Other cancers	87 (8.1)	143 (11.8)	2.07 (1.45, 2.96)^b^

a ORs presented in the table were derived from a main effect multivariate model including age at interview, place of birth, education level, past history of lung diseases, intake of non-orange related fruit, residential radon exposure, smoking status, smoking pack-years, and a family history of overall cancers.

b ORs were adjusted for age at interview, place of birth, education level, past history of lung diseases, intake of non-orange related fruit, residential radon exposure, smoking status, and smoking pack-years.

Table 2 shows the effects of alcohol consumption in the subgroups of never, former, and current smokers. A weak and positive association between alcohol consumption (occasional or frequent users) and lung cancer was observed in never smokers, while the strong and significant joint effects between alcohol and smoking (former or current smokers) were mostly be attributed to the effects of smoking: the OR for frequent alcohol consumption was approximate 1.51 (3.49/2.31) among the former smokers and 1.20 (10.98/9.13) among the current smokers compared with the never drinkers in each corresponding category. Since there was no significant multiplicative-scale interactions between smoking status and alcohol consumption on lung cancer risk (p>0.9), smoking was justified as a potential confounder which was thus to be adjusted for in the multivariate analyses. We estimated the synergy index for the joint effect of alcohol and smoking on lung cancer risk, and it was close to one (SI = 1.17, 95%CI: 0.78–1.82), suggesting no interaction on the additive scale. We combined never and occasional alcohol users into ‘nonusers’ category because their effects in current, former or never smokers were similar (Table 2).

**Table 2:** Estimating the odds ratio (OR) and 95% confidence interval of alcohol consumption with lung cancer risk in never, former, and current smoking Chinese men, 2004–2006^a^.

Frequency of alcoholconsumption	Never smoking		Former smoking		Current smoking		Pvalue^d^
	Referents/Cases	Odds ratio	Referents/Cases	Odds ratio	Referents/Cases	Odds ratio	
Ordinal category^b^							0.911
Never	333/69	1.00	138/113	2.31 (1.52–3.52)	70/263	9.13 (5.88­14.20)	
Occasional	110/34	1.31 (0.80–2.14)	90/79	2.19 (1.38–3.46)	42/156	9.18 (5.59–15.08)	
Frequent	90/28	1.37 (0.80–2.32)	124/147	3.49 (2.30–5.29)	62/312	10.98 (6.96–17.33)	
Binary category^b^							0.902
Nonusers^c^	443/103	1.00	228/192	2.08 (1.47–2.95)	112/419	8.44 (5.81–12.27)	
Frequent	90/28	1.26 (0.76–2.09)	124/147	3.22 (2.18–4.74)	62/312	10.12 (6.59–15.55)	

a Missing data were not included in the analyses.

b Variables included in the models were age, place of birth, education level, family cancer history, past history of lung diseases, intake of non-orange related fruit, residential radon exposure, and smoking pack-years.

c Nonusers of alcohol included both never and occasional users of alcohol.

d P value for multiplicative-scale interaction (i.e., likelihood ratio test for interaction) between exposures of interest.

Compared with nonusers of alcohol who had no family cancer history, the smoking (and other potential confounders) adjusted OR for all the frequent alcohol users with family cancer history was 4.22 (95%CI: 2.46–7.23, 103 cases) (Table 3). The smoking (and other potential confounders) adjusted OR for the alcohol nonusers with family cancer history and the frequent alcohol users without family cancer history was 1.95 (95%CI: 1.37–2.78, 134 cases) and 1.24 (95%CI: 0.95–1.63, 308 cases), respectively. We observed a significant synergy index of 2.70 (95% CI: 1.11–7.87) between alcohol consumption and family cancer history among all participants. Further stratified analyses according to smoking status showed that the OR for the joint effect of alcohol and family cancer history among never smokers (4.85, 95% CI: 1.73–13.59, 9 cases) was slightly higher than that of the smokers (3.84, 95% CI: 2.04–7.21) (Table 3). The joint effect between alcohol and family cancer history tended to be consistent with an additive interaction, though the SI among never smokers was not statistically significant and relatively low (SI = 1.88, 95% CI: 0.44–8.00). There was no significant multiplicative-scale interaction between alcohol consumption and family cancer history on the risk of lung cancers for all the participants (p = 0.09) as well as in the subgroups of never smokers (p = 0.51) and smokers (p = 0.08).

**Table 3:** Estimating the separate and joint effects of alcohol consumption and family cancer history on the risk of all lung cancers and adenocarcinoma among Hong Kong men in 2004–2006, stratified by smoking status^ab^.

	Negative family cancer history		Positive family cancer history				
Alcohol consumption	Referents/Cases	OR (95% CI)	Referents/Cases			OR (95% CI)	
All participants^c^							
Nonusers	557/474	1.00	107/134			1.95 (1.37–2.78)	
Regular	198/308	1.24 (0.95–1.63)	27/103			4.22 (2.46–7.23)	
Synergy index						2.70 (1.10–6.61)	
Never smokers^d^							
Nonusers	322/58	1.00	62/30			3.06 (1.72–5.44)	
Regular	63/14	0.99 (0.50–1.98)	12/9			4.85 (1.73–13.59)	
Synergy index						1.88 (0.44–8.00)	
Ever smokers^c^							
Nonusers	235/416	1.00	45/104			1.51 (0.98–2.34)	
Regular	135/294	1.21 (0.90–1.64)	15/94			3.84 (2.04–7.21)	
Synergy index						3.92 (1.08–14.28)	
Adenocarcinoma^c^							
Nonusers	557/168	1.00	107/55	1.92 (1.26, 2.94)			
Regular	198/102	1.31 (0.93, 1.84)	27/44	4.66 (2.56, 8.50)			
Synergy index					2.98 (1.10–10.63)		

Abbreviation: OR = odds ratio;

95% CI = 95% confidence interval.

a Missing data were not included in the analyses.

b Nonusers of alcohol in the table included both never or occasional users of alcohol.

c Variables included in the models were age, place of birth, education level, past history of lung diseases, intake of non-orange related fruit, residential radon exposure, smoking status, and smoking pack-years.

d Variables included in the models were age, place of birth, education level, past history of lung diseases, intake of non-orange related fruit, residential radon exposure.

A similar OR for the joint effect of alcohol and family cancer history was observed (4.66, 95% CI: 2.56–8.50, 44 cases) when the analyses were restricted to the adenocarcinoma (Table 3), with a comparable SI of 2.98 (95% CI: 1.10–10.63). No significant multiplicative-scale interaction was suggested between alcohol consumption and family cancer history on the risk of adenocarcinoma (p = 0.09); however, further analyses according to smoking status for adenocarcinoma were hampered by too few never smoker cases.

## Discussion

This case-referent study revealed that the joint effect between frequent alcohol consumption and family cancer history was likely to be consistent with a synergistic additive interaction. Our study did not support the findings from a previous study that smoking had modified the effect of alcohol consumption on lung cancer risk [25], while cigarette smoking in our study was evident to play a role of a confounder for the association between alcohol and lung cancer.

Residual confounding of cigarette smoking might be a concern when interpreting the effect of alcohol, as the habits of alcohol consumption and smoking are usually closely correlated [9]; in our study, the correlation coefficient between smoking and alcohol consumption was 0.21. We carefully addressed the potential confounding effect of smoking by including both smoking status and smoking pack-years into the models. The OR for alcohol consumption remained unchanged when the smoking pack-years was replaced by daily amount of cigarettes consumed and years of smoking (data not shown). We further addressed the possible residual confounding from smoking by comparing ‘years since smoking cessation’ and there were no obvious differences among different categories of alcohol users. We observed a positive but weak association between regular alcohol consumption and lung cancer, and that association was independent of smoking status.

One of the strengths of this study is that, besides detailed smoking data, almost all other known possible confounding factors (e.g., residential radon exposure, dietary factors, etc) were adjusted in our multivariate analyses. To the best of our knowledge, only one previous study reported an interactive effect (using a likelihood ratio test for interaction only) between alcohol and smoking on lung cancer [25] and there has been no report about the joint effect between alcohol and family cancer history. Our study was the first to systematically evaluate whether the joint effect between alcohol and smoking or family cancer history conformed to a multiplicative or additive risk effect.

Results from our study demonstrated that a significantly increased OR was only restricted to frequent alcohol users who had family cancer history after smoking and other potential confounders were carefully adjusted, consistent with a synergistic additive interaction. Animal studies had shown that ethanol itself is not a carcinogen [26], but its metabolite acetaldehyde is highly toxic, carcinogenic and mutagenic which can interfere with the synthesis and repair of DNA [27], [28] and might increase the P53 gene mutations during the duplication of DNA [29]. As proposed by Poschl and Seitz [30], chronic alcohol consumption may also cause an induction of cytochrome P-4502E1 (CYP2E1) which can enhance activation of various procarcinogens present in alcoholic beverages. Moreover, alcohol may increase the uptake of environmental carcinogens from tobacco smoke by inducing activation of CYP2E1 or through damaged cell membranes by direct effect of alcohol to enhance the risk of lung cancer [30].

In a recent case-referent study (referents were derived from a community setting) among 400 Indian men (1∶1 matching), Shah and his colleagues found that alcohol consumption might have interacted with CYP1A1 genotypes to increase the risk of squamous cell lung cancer, and an interaction between alcohol consumption and genetic factors was indicated in the development of lung cancer [14]; however, residual confounding effect of smoking and the effects from other potential confounding factors was a concern of that Indian study because only age and smoking status (smokers vs. nonsmokers) were adjusted in the model. Our data only allowed us to estimate the ORs for the adenocarcinoma according to alcohol status and family cancer history, and we found that the risk estimates for adenocarcinoma were comparable to all lung cancers combined. It is also interesting to look into the associations between alcohol and other cell types of lung cancer; however, our study has limited power to carry out an extensive subgroup analysis by histology.

To further elucidate the potential residual confounding effect from smoking in studying the separate effect of alcohol consumption as well as the joint effect with family cancer history, we repeated the data analyses in never smokers and smokers separately. We did not observe an excess risk of lung cancer for frequent alcohol consumption among never smokers who had no family cancer history (though the power is very limited), but we found a slightly increased OR of alcohol consumption among all the participants (1.24) and the subgroup of smokers (1.21). Our study indicated that a careful adjustment of smoking may not be able to completely rule out the residual confounding of smoking, given an overwhelming effect of smoking on lung cancer risk. Hence, future larger analytic studies including more never smoking lung cancer cases are essentially needed.

Selection bias could be a concern because our lung cancer cases (96%) and referents (48%) had different participant rates; nevertheless, we chose the community referents matched for the district of residence of the cases, which increases the comparability between the cases and the community referents in socioeconomic background. Recall bias on reporting family cancer history was also a concern but it was likely non-differential which would lead to an underestimation of the risk estimates. We are aware of the crude measurement of alcohol intake of this study and hence the mechanisms involved in the observed interaction should be further explored in future studies with more reliable dose quantification of alcohol consumption and more never smoking cases. Since we did not obtain information on alcohol consumption and smoking among family members, this study could not allow us to disentangle to what extent the association between lung cancer and family cancer history could be attributable to shared environmental exposures of family members or shared genetic susceptibility.

In conclusion, this study revealed a possible synergistic additive interaction between frequent alcohol consumption and familial susceptibility for lung cancer risk, whereas the independent effect of alcohol (if it is present) should be weak. However, these observed possible associations need to be confirmed by future larger analytic studies with more never smoking cases.
